# 38 Spectacular improvement of refractory calcaneal enthesitis with anti TNF alpha

**DOI:** 10.1093/rheumatology/keac496.034

**Published:** 2022-10-07

**Authors:** Y Sadeg, A Ferhat, H Hafirassou, C Dahou-makhloufi

**Affiliations:** Rheumatology Department of Bab-El-Oued University Hospital, Algiers, Algeria

## Abstract

**Background:**

Spondyloarthritis is a heterogeneous group of chronic inflammatory rheumatism, of which enthesitis is the most common. Achilles involvement is frequent and can be disabling, resisting various treatments (non-steroidal anti-inflammatory drugs and local treatment). Only anti TNF alpha drugs are effective.

We report the case of a patient with extremely painful achilles enthesitis causing functional disability who responded dramatically to anti TNF alpha.

**Observation:**

Child A.N, 13 years old, followed for juvenile idiopathic arthritis in the form of psoriatic arthritis evolving since the age of 4 years, treated with methotrexate at a rate of 7.5 mg/week, well balanced for 7 years.

Three years later, the patient consulted us for disabling right inflammatory heel pain with the impossibility of putting his shoes on.

The clinical examination revealed a painless position with the inability to put the heel on the ground; difficulty in walking requiring the use of crutches; pain on palpation of the Achilles tendon up to its insertion on the calcaneus, with painful limitation of dorsiflexion. Skin and mucous membrane examination revealed psoriasis lesions on the dorsum of the foot and on the nails.

Laboratory tests: CBC without abnormality, ESR= 25mmH1 CRP =12mg/l

X-ray of the rear feet: no abnormalities.

Ultrasound of the right ankle: enthesitis taking colour doppler at the insertion of the Achilles tendon with erosion within the calcaneus.

Magnetic resonance imaging of the right ankle: medullary oedema in the calcaneus with hyper signal on the DP sequence associated with retro calcaneal bursitis.

The patient was put on NSAIDs without improvement, two ultrasound-guided cortisone injections were performed, followed by total failure. Treatment with an anti TNF alpha (Etanercept 0.8 mg/kg/week 25 mg) was started, the improvement was spectacular after 8 weeks with disappearance of the pain, including when standing, walking and putting shoes on. On the ultrasound (disappearance of the retro calcaneal bursitis and negativation of the doppler signal) no recurrence was noticed.

**Conclusion:**

Enthesitis is an essential lesion in spondyloarthritis. Generally resistant to conventional DMARDs, anti TNF alpha is a treatment of choice.

